# Machine Learning-Based Boosted Regression Ensemble Combined with Hyperparameter Tuning for Optimal Adaptive Learning

**DOI:** 10.3390/s22103776

**Published:** 2022-05-16

**Authors:** Joseph Isabona, Agbotiname Lucky Imoize, Yongsung Kim

**Affiliations:** 1 Department of Physics, Federal University Lokoja, P.M.B 1154, Lokoja 260101, Nigeria; joseph.isabona@fulokoja.edu.ng; 2 Department of Electrical and Electronics Engineering, Faculty of Engineering, University of Lagos, Akoka, Lagos 100213, Nigeria; aimoize@unilag.edu.ng; 3 Department of Electrical Engineering and Information Technology, Institute of Digital Communication, Ruhr University, 44801 Bochum, Germany; 4 Department of Technology Education, Chungnam National University, Daejeon 34134, Korea

**Keywords:** 5G performance measurement, throughput data, adaptive learning, machine learning, random forest, hyperparameter tuning, optimization, least-squares boosting

## Abstract

Over the past couple of decades, many telecommunication industries have passed through the different facets of the digital revolution by integrating artificial intelligence (AI) techniques into the way they run and define their processes. Relevant data acquisition, analysis, harnessing, and mining are now fully considered vital drivers for business growth in these industries. Machine learning, a subset of artificial intelligence (AI), can assist, particularly in learning patterns in big data chunks, intelligent extrapolative extraction of data and automatic decision-making in predictive learning. Firstly, in this paper, a detailed performance benchmarking of adaptive learning capacities of different key machine-learning-based regression models is provided for extrapolative analysis of throughput data acquired at the different user communication distances to the gNodeB transmitter in 5G new radio networks. Secondly, a random forest (RF)-based machine learning model combined with a least-squares boosting algorithm and Bayesian hyperparameter tuning method for further extrapolative analysis of the acquired throughput data is proposed. The proposed model is herein referred to as the RF-LS-BPT method. While the least-squares boosting algorithm is engaged to turn the possible RF weak learners to form stronger ones, resulting in a single strong prediction model, the Bayesian hyperparameter tuning automatically determines the best RF hyperparameter values, thereby enabling the proposed RF-LS-BPT model to obtain desired optimal prediction performance. The application of the proposed RF-LS-BPT method showed superior prediction accuracy over the ordinary random forest model and six other machine-learning-based regression models on the acquired throughput data. The coefficient of determination (Rsq) and mean absolute error (MAE) values obtained for the throughput prediction at different user locations using the proposed RF-LS-BPT method range from 0.9800 to 0.9999 and 0.42 to 4.24, respectively. The standard RF models attained 0.9644 to 0.9944 Rsq and 5.47 to 12.56 MAE values. The improved throughput prediction accuracy of the proposed RF-LS-BPT method demonstrates the significance of hyperparameter tuning/optimization in developing precise and reliable machine-learning-based regression models. The projected model would find valuable applications in throughput estimation and modeling in 5G and beyond 5G wireless communication systems.

## 1. Introduction

Effective data processing and analysis have become a huge task due to the upsurge in massive data collection from various wireless communication devices and cellular networks [1]. Massive data can be classified into two main parts, right and raw data. The present generation of cellular broadband networks, such as 4G and 5G and including the envisioned 6G, can be improved if the correct data are extracted from the available massive data and mined resourcefully to reveal the actual quality status [2]. Predictive mining of such data can also aid in optimal planning and optimization of such system networks due to the emergence of artificial intelligence and machine learning, which enable such interconnected processes to be achieved through robust and efficient data analytics [3,4,5].

In recent decades, machine-learning-based regression models have emerged as vital models in carrying out prognostic modeling and analysis of relevant datasets in all fields of physical sciences, medical science, and engineering domains [6,7,8,9,10,11,12,13,14,15]. One notable advantage of machine learning over other data analytic techniques is its ability to identify patterns and trends with ease and high accuracy adaptively. Another significant advantage is its capability to automate and solve many complex decision-making tasks.

Several machine learning techniques have been explored [16,17,18,19,20,21]. Some of the key ones include random forest (RF) [22,23], support vector machine (SVM), neural networks (NNs), K-nearest neighbor (KNN), and the Gaussian process (GP). Among these techniques, RF stands out owing to its distinctive adaptive learning and predictive modeling capability [1,24,25]. RF is suitable for adaptive regression-based learning and the effective classification of extensive data. It can handle both simplified and complex datasets containing real or contiguous values. Interestingly, RF is less sensitive and robust to outliers. RF can be explored for prognostic estimation and mapping high-dimensional data. These exceptional and matchless capabilities of RF have led to its increasing popularity for constant usage in diverse research fields, including sciences and engineering. However, the performance accuracy of regression-learning-based RF models is influenced mainly by the input data, training algorithm, and regulating hyperparameters [6,7,8]. One of the hyperparameters is the tree number and value. The selection of tree numbers that are too large or small can cause overfitting, poor generalization, slow implementation, and poor real-time predictions.

Many attempts have been made to handle RF hyperparameter tuning problems in the literature to boost its predictive application performance. In the literature [6,7], the authors examined how to determine the optimum number of RF trees. Particularly in [8], the work investigated how to optimally employ the RF feature set size for robust regression analysis of 56 different datasets. The researchers found that the optimal size tends to be comparatively small if the dataset features are correlated and vice versa. In [9], the influence of tree size was also studied. The study developed a new ensemble technique for refining and growing trees in depth for the RF model. In [10], a weighted voting technique was introduced into a random forest algorithm to enhance its application for employee turnover prediction. The authors in [11] added the self-organizing mapping technique to the RF modeling process to boost pediatric fracture healing time predictive analysis. In [12], the authors engaged the class weight RF algorithm to solve and analyze medical class imbalance data effectively. In the context of classification, References [13,14,15] examined and reported the influence of feature set size on RF prognostic estimation performance. A summary of related works [26,27,28,29,30,31,32,33] which employ machine-learning-based models, capturing their focus and coverage, key limitations, and a comparison with this paper, is presented in Table 1.

In view of the preceding literature, there is no existing work that reports a machine-learning-based boosted regression ensemble combined with hyperparameter tuning for optimal adaptive learning. To this end, this paper proposes a random forest machine-learning-based model combined with a least-squares boosting algorithm and Bayesian hyperparameter tuning to boost its predictive application performance. The proposed regression model is termed the RF-LS-BPT model. A detailed application of the proposed RF-LS-BPT model to real-time throughput data acquired at different user equipment terminal locations in 5G mobile broadband cellular networks was investigated. Our proposed RF-LS-BPT model offers a new hybridized predictive modeling method to help network operators and engineers regularly conduct improved extrapolative analysis of different cellular network data for planning and management purposes.

The foremost contributions of this research paper are highlighted as follows:

- We first give a detailed statistical analysis of the acquired throughput data through performance status reporting at the different user equipment terminal locations with respect to the tested communication distances from the transmitter.
- We provide performance benchmarking of adaptive learning capacities of different key machine-learning-based regression models with the choice regression model, which is the random forest.
- We propose an RF-LS-BPT regression model for improved dataset predictive modeling and learning.
- The proposed RF-LS-BPT regression model was applied in detailed, accurate throughput data modeling and learning using different performance indicators.

The remaining part of this paper is structured into four sections as follows. Section 2 contains the related work and background information. Section 3 offers the machine-learning-based boosted regression method, 5G throughput measurement campaign, and the proposed RF-LS-BPT algorithm and implementation process. Section 4 focuses on the results and discussion. Finally, the conclusions are drawn in Section 5.

## 2. Theoretical Background

First, the general concept of random forest is described, and the exploratory data analysis (EDA) method applied is highlighted in this section. The mathematical description of the RF regression model and least-squares boosting (LS-Boost) is also broached.

### 2.1. Random Forest (RF)

The RF is an ensemble of decision-tree-based machine learning methods. It was proposed in [34] by Breiman to address both data classification and regression problems. It operates by growing and assembling a host of self-regulating decision trees to solve complex real-world problems. While the trees grow, the data are shared using a principle in several steps. The performance accuracy of regression-learning-based RF models is primarily influenced by the input data, training algorithm, and regulating hyperparameters [35]. Here, ‘hyper’ indicates top-level parameters that can be explored to regulate the machine learning process and produce better results. Some of the vital RF hyperparameters include the decision tree number, tree type, and the feature set size (number of features), all of which control performance. Hyperparameter tuning or optimization is a robust method of identifying and finding the best feasible values of hyperparameters for a machine learning model to attain the desired resultant modeling outcome. Popular hyperparameter tuning algorithms in the literature include random search, grid search, and Bayesian optimization search [36,37].

Generally, many factors impact on predictive modeling capacities of machine-learning-based models and methods, especially the surrogate types such as the RF, SVM, DT, GPR, and NN. These include the learning rate, tree number, training algorithm, and hyperparameter tuning algorithm. In [7], the authors concentered on how to explore different key RF modeling parameters such as tree number (size) and related features to effectively mine different datasets. Particularly in [8], the researchers’ interest was how to optimally implore the RF feature set size to conduct a robust regression analysis of large datasets.

In this study, an integrated exploratory approach was taken to examine some of the aforementioned factors in RF predictive modeling performance capacity. Our exploratory approach considers the integration of a random forest (RF)-based machine learning model combined with a least-squares boosting algorithm and Bayesian hyperparameter tuning method for real-time extrapolative data analysis.

### 2.2. Exploratory Data Analysis Procedure

The exploratory data analysis (EDA) method [38] was utilized in this study. It is a systematic method of investigating and analyzing datasets to discover patterns and ensure that valid results are produced according to desired goals. A regression-based machine learning model is expected to learn a dataset adaptively, thereby identifying and bringing out the relationships between data input values and targeted output response during training. Effective predictive data processing is part of a critical step in discovering patterns in data.

### 2.3. The RF Regression Model and Least-Squares Boosting (LS-Boost)

In broad mathematical terms, an RF is a special predictor whose main constituents are built on randomized tree ensembles ${\left\{Y\left(x;\Theta_{a_{t}},R_{n}\right)\right\}}_{1\leq a\leq A}$. The sequence ${\left\{\left(\Theta_{a_{t}}\right)\right\}}_{1\leq a\leq A}$ encloses the random variables Θ that regulate the probabilistic mechanism wherein each tree is built.

For a finite tree number, *A*, the RF estimate can be expressed as (1):

(1) $$Y_{A}\left(X;\Theta_{1_{t}},\Theta_{2_{t}}\ldots ,\Theta_{A_{t}}R_{n}\right):=\frac{1}{A}\sum_{a=1}^{M}Y\left(X;\Theta_{a_{t}}R_{n}\right)$$

For an infinite tree number (i.e., *M* is sufficiently large), the RF estimate turns to (2):

(2) $$Y_{A}\left(X,R_{n}\right):=E_{\Theta }\left[Y\left(X;\Theta_{a_{t}}R_{n}\right)\right]$$

where $E_{\Theta }$ indicates the expectation value in correspondence with Θ.

The individual tree predictor can be defined by (3):

(3) $$Y_{}\left(X\right):=\sum_{}^{}E_{\Theta }\left[W_{i}\right]Y_{i}{}_{}$$

where $W_{i}:=\frac{1_{\left(x\in t\right)}}{N(t)}$ indicates the averaging weight.

Consider a data training sample ${ℜ}_{a}=\left[\left(Y_{1},X_{1}\right),\left(Y_{2},X_{2}\right)\right],\ldots ,\left[\left(Y_{A},X_{A}\right)\right]\;\mathrm{of}\;{\left[0,1\right]}^{p}\;$, of real-valued random variables. The leading objective is to predict the target response Y connected to the random variable, X, employing a regression function f(x)=E[|Y|X=x]. Accordingly, the loss function which defines the mean squared error (MSE) can be estimated using (4):

(4) $$L(Y,f(X_{i}))={\sum_{i=1}^{K}\;\left(Y_{1}-f(X_{i})\right)}^{2}$$

Owing to the hypothetical bias and variance issues, the fitted model and the resulting predicted outcome may severely suffer from underfitting or overfitting problems, leading to a high error between the targeted response and the estimated variables. In order to address such drawbacks, the inconsistency of $f\left(X_{1}\right)$ in Equation (4) needs to be placed under control by employing the bagging (Bag) or least-squares boosting (LS-Boost) algorithm. This paper considers the LS-Boost algorithm but employs bagging to benchmark the results. In the LS-Boost algorithm, hundreds or more weak learners (trees) are engaged for training, and it iteratively updates the error to become a strong learner [34,38]. At every iteration step, the ensemble fits in a fresh learner. The MSE are expressed in Equation (4).

## 3. The Proposed Machine-Learning-Based Boosted Regression Ensemble Combined with Hyperparameter Tuning

This section presents detailed information on the entire procedure engaged to achieve the research aim. Particularly, the method of 5G throughput data collection, the proposed RF-LS-BPT implementation algorithm, and its implementation process are provided in this section.

### 3.1. 5G Throughput Measurement Campaign

The current study utilized field measurements taken in diverse urban environments in the United States to test and validate the proposed learning-based models. The field measurements were taken to assess the commercial 5G performance on smartphones and made available online [31]. The 5G networks of three carriers in three US cities were examined. Specifically, a systematic analysis of the various mechanisms used for handoff in the 5G network was explored. The impact of these handoff mechanisms on network performance was also feasibly explored to determine whether the location and other dynamic environmental conditions can be used to predict network performance.

Additionally, the performance of the app in terms of web browsing, HTTP download, and volumetric video streaming over 5G was critically examined. The experiments, which consume over 15 Tb data, were carried out over T-Mobile, Sprint, and Verizon 5G networks. Verizon offers operational mm-wave-based 5G services to subscribers in the investigated environments where dense 5G base stations are deployed. T-Mobile employs mm-wave, while Sprint uses a mid-band frequency at 2.5 GHz. In the field measurements, the authors captured about 6.8 million data points obtained from the 5G coverage in downtown Minneapolis, USA [31].

Two types of commercially available off-the-shelf (COTS) 5G-capable smartphones were used in the experiments. These were the Motorola Moto Z3 and Samsung Galaxy S10 5G (SM-G977U). For brevity, these are described as MZ3 and SGS10, respectively. The SGS10 uses an in-built 5G radio, while the MZ3 uses an external 5G mod to access 5G networks. The mobile device used is the SGS10, and it is 4G- and 5G-compatible, allowing comparison on the same device. Four locations were considered for the experiments carried out on Verizon’s network [31]. Typically, the locations are a good representative of open/crowded spaces, low/high buildings, indoor/outdoor environments, and more. The experimentation was conducted using a Microsoft Azure server to achieve the highest statistical 5G throughput. The server also helps to achieve approximately 3 Gbps throughput.

### 3.2. The Proposed RF-LS-BPT Process

**Figure 1:**
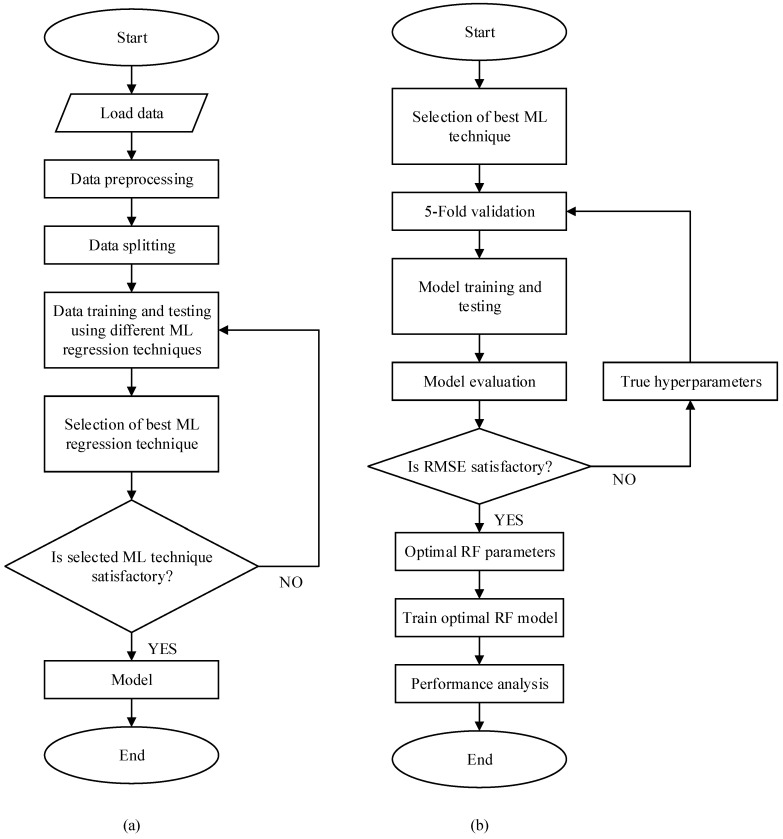
(**a**) Flowchart for the Proposed RF-LS-BPT model; (**b**) the RF-LS-BPT model and its hyperparameter tuning implementation process.

The entire RF-LS-BPT process, which is revealed using the flowchart in Figure 1 and its stepwise implemented method using MATLAB, is outlined as follows. Also, the RF regression with least-squares boost (LS-Boost) is given in Algorithm 1.

- Load the throughput datasets into MATLAB.
- Examine the datasets to obtain relevant insights.
- Presence of correlated features.
- Missing values and outliers.
- Preprocess the datasets to cater for the identified missing values and outliers.
- Transform the datasets RF-LS-BPT modeling format.
- Split the datasets into two, with 0.3 portions for testing and 0.7 portions for training.
- Engage the default RF ensemble fitting tool in MATLAB for the data training and testing.
- Evaluate the default RF ensemble fitting through data training and testing.
- Choose an appropriate RF aggregation technique. LS-Boost was chosen here.
- Identify the most relevant RF hyperparameters.
- Determine optimal values of the RF hyperparameters using the optimization option I MATLAB (‘OptimizeHyperparameters’, ‘auto’), which is based on the Bayesian optimization process.
- Optimize the RF Regression ensemble results using the cross-validation process.
- Build the final RF-LS-BPT model combing the LS-Boost algorithm with tuned optimal RF hyperparameter values.
- Engage the resultant RF-LS-BPT model on the entire throughput quality datasets.
- Test the resultant RF-LS-BPT using a 0.3 portion of the data and new data.
- Assess and report the predictive performance of the resulting RF-LS-BPT model.

**Algorithm 1:** Also, The RF Regression with least-squares boost (LS-Boost) is given in Algorithm 1.**Input:** Training set:$\left[\left(y_{1},x_{1}\right),\left(y_{2},x_{2}\right)\right],\ldots ,\left[\left(y_{K},x_{K}\right)\right]$. Learning rate value, v and Tree number, A, obtained through Bayesopt, Loss function, $L(y,f(x_{i})).$ **Output:** Regression mode, $F_{a}(x),f(x_{i})=\bar{y}$ **Training Process:** For a=1 to A, do: $f(x_{i})={\left(\bar{y}-F_{a-1}(x)\right)}_{i}^{k}$ $\mathrm{Train}\;B_{a}(x)\;\mathrm{using}\;{\left(x,\bar{y}\right)}_{i}^{k}$ $p_{a}(x)={\arg\;\min}_{p}\;{\sum_{}^{}\left[\bar{y}-pB_{a}\left(x\right)\right]}^{\;2}$ $F_{a}(x)=F_{a-1}(x)+v\sum_{a=1}^{A}p_{a}B_{a}\left(x\right)$ **End**

### 3.3. Key Evaluation Metrics

The mean absolute error (MAE) [39] given in (5), the normalized mean squared error (NRMSE) given in (6), the coefficient of determination (Rsq) given in (7), and the percentage error (PE) are the five key evaluation metrics used in this paper to examine the performance of the RF-LS-BPT method. The proposed method is better if the MAE and NRMSE values are low but have higher Rsq values.

(5) MAE=1K∑i=1K|ymi−ypi|

(6) NRMSE=1K∑i=1K(ymi−ypi)2/(ypi(max)−ypi(min))

(7) $$R^{2}=1-\left(\frac{\sum_{i=1}^{K}{\left(y_{mi}-y_{pi}\right)}^{2}}{\sum_{i=1}^{K}{\left({\overset{⌢}{y}}_{mi}-y_{pi}\right)}^{2}}\right)$$

## 4. Results and Discussion

Detailed results and discussion are contained in this section. All computations, coding, implementation, and graphics were conducted using MATLAB software environment with the aid of an HP laptop (Elitebook) with an Intel^®^ Core^™^ i3-10110U and Intel^®^ Turbo Boost Technology, 4 MB L3 cache, 2 cores was used. First, in this section, we start by revealing the status of the acquired 5G throughput qualities attained at close communication distances of 25, 50, 75, 100, and 160 m between the transmitter and UET. This is followed by results and discussion on the throughput data training and testing accuracy achieved using seven machine learning models with their default parameters. Also contained in this section are throughput data training and testing results achieved using the proposed RF-LS-BPT model versus the standard RF modeling approach, plus results on throughput data training and testing using LS-boosting and bagging.

### 4.1. Throughput Quality Status Analysis

**Figure 2:**
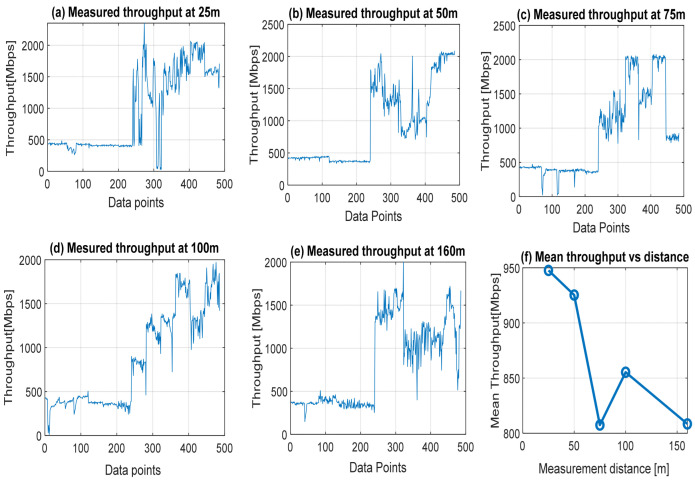
Measured throughput qualities attained at *close* communication distances of 25, 50, 75, 100, and 160 m between the transmitter and UET.

The throughput quality remains an exclusive higher-layer performance indicator for assessing data transmission quality and integrity in mobile broadband networks. Remarkably, the actual throughput quality at the UET can be influenced by critical factors such as user location and communication distance from the transmitter. The user data throughput expresses the speed at which a user can reliably send data and receive the same at the user equipment terminal (UET). It also expresses the quantity of data in bits per second (bps) conveyed and delivered over the cellular network within a specific period. The graphs in Figure 2 display the measured throughput qualities attained at close communication distances of 25, 50, 75, 100, and 160 m between the transmitter and UET. All the graphs show that the network experiences low throughput quality in the range of 50 to 100 Mbps at first user download before experiencing upward but fluctuating quality improvement as the user stays in the network. The low throughput quality experienced at the UET shows that the network has serious delay problems during the initial network log-in. The general fluctuations in throughput quality across the various measurement distances can be attributed to several influencing factors, which include network propagation environment, user location, communication distance, the asymmetry between upload and download rates, available channel bandwidth, network traffic load, propagation channel conditions, signal quality, signal coverage, and modulation/coding scheme [40].

Throughput quality status in terms of maximum, minimum, and mean throughput quality values attained at the various distances are summarized in Table 2. For maximum quality, about 2350, 2080, 2070, 1970, and 1990 Mbps values were attained at 25, 50, 75, 100, and 160 m UET location distances. A close look at the values shows that the maximum quality value attained by the UET degrades as communication distance increases for the connecting transmitter. This result confirms that user equipment location regarding communication distance from the transmitter antenna is a major factor influencing the quality level received. Overall, the mean throughput quality attained at the UET is quite low compared to the at least 1000 Mbps quality value envisioned for 5G broadband networks, even at such close distances.

### 4.2. Throughput Data Training and Testing Using Different Machine Learning Models with Their Default Parameters

**Figure 3:**
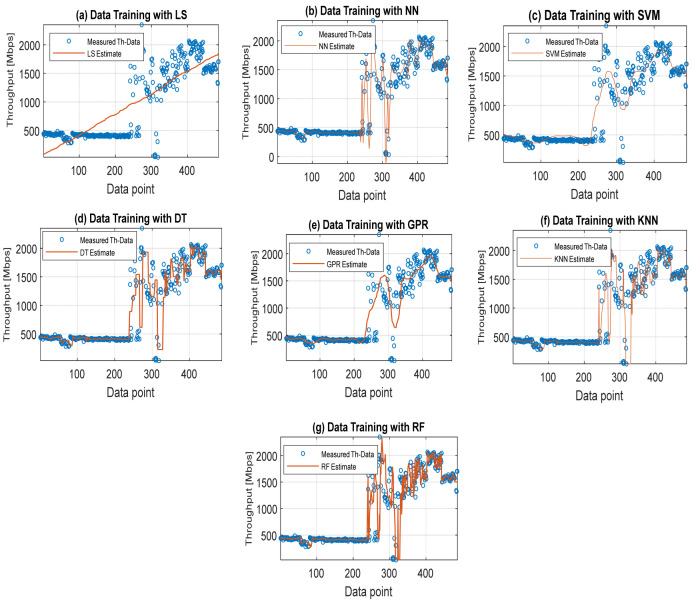
Throughput quality comparison of different machine learning models. (**a**) Least-squares (LS). (**b**) Neural networks (NNs). (**c**) Support vector machine (SVM). (**d**) Decision tree (DT). (**e**) Gaussian process regression (GPR). (**f**) K-nearest neighbor (KNN). (**g**) Random forest (RF).

**Figure 4:**
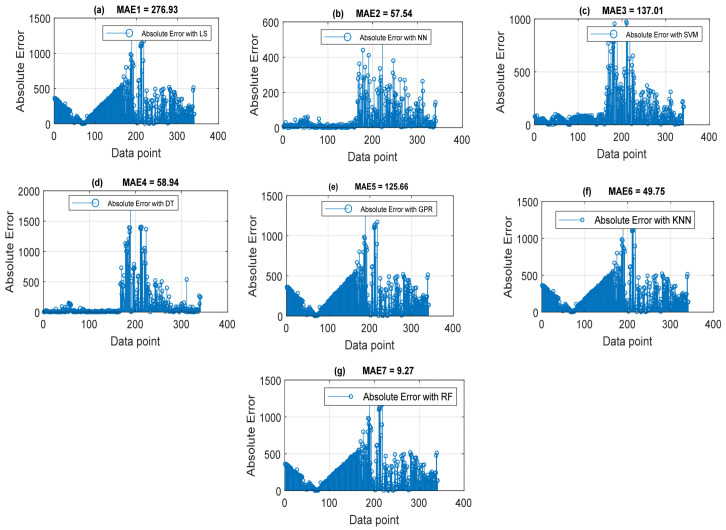
Throughput quality prediction accuracy with MAE attained by the different machine learning models. (**a**) Least-squares (LS). (**b**) Neural networks (NNs). (**c**) Support vector machine (SVM). (**d**) Decision tree (DT). (**e**) Gaussian process regression (GPR). (**f**) K-nearest neighbor (KNN). (**g**) Random forest (RF).

**Figure 5:**
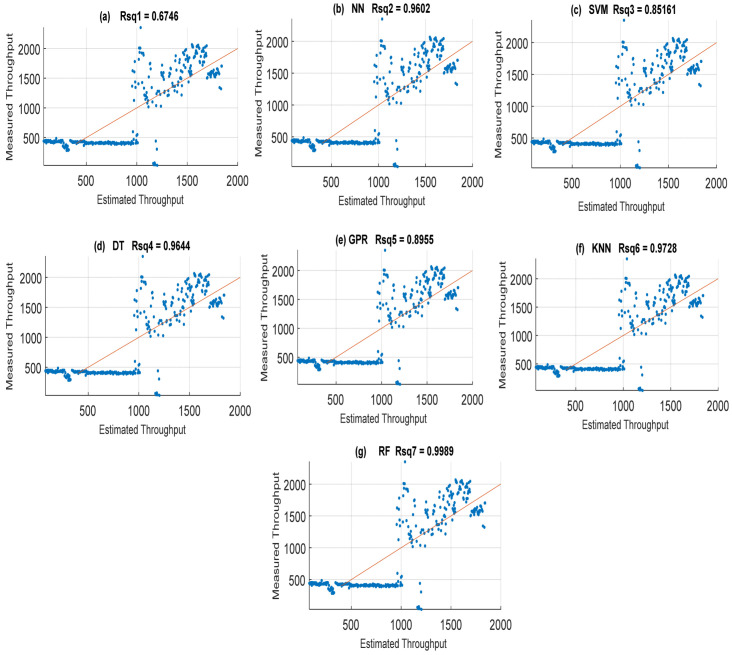
Throughput quality prediction accuracy with Rsq attained by the different machine learning models on throughput quality. (**a**) Least-squares (LS). (**b**) Neural networks (NNs). (**c**) Support vector machine (SVM). (**d**) Decision tree (DT). (**e**) Gaussian process regression (GPR). (**f**) K-nearest neighbor (KNN). (**g**) Random forest (RF).

In addition to hyperparameters, machine learning models have their default parameters internally built for specific tasks. While the default parameters are inevitably used to learn, hyperparameters are objectively set by the user to guide the learning process optimally. Here, five key machine learning models with default parameter regression settings were first engaged for throughput data training and testing. The machine learning models are the multi-layer perceptron neural network (MLP-NN), random forest (RF), support vector machine (SVM), K-nearest neighbor (KNN) model, Gaussian process regression (GPR), and decision tree (DT). The generalized least-squares (GLS) model was also employed in the regression process. The main aim of using other machine learning methods is also to assess their adaptive learning capability with the choice of RF method. Shown in Figure 3 is a plot displaying the throughput quality comparison of different machine learning models. The prediction performance of the individual methods explored in terms of their accuracy using MAE and R-Squared (Rsq) is shown in Figure 4 and Figure 5. The MAE and Rsq values attained for GLS, MLP-NN, RF, SVM, KNN, GPR, and DT were 276.96, 57.54, 137.01, 58.94, 125.66, 276.96, and 9.27 dB and 0.6746, 0.9602, 0.8642, 0.9644, 0.87.55, 0.9728, and 0.9989, respectively. The RF regression model achieved the lowest MAE value of 9.27 dB and the best 0.9998 Rsq value from these throughput data training results. Similar superior prediction efficiency results were obtained with the RF regression model when engaged for throughput data testing. Still, the results are excluded here for the sake of brevity. The better prediction efficiency of RF could be due to its robust ability to handle large dimensionality datasets efficiently with high precision. On the other hand, the GLS attained the worst results because of its poor performance in handling stochastic datasets with high variance and large dimensionality [41,42].

### 4.3. Throughput Data Training and Testing Using Proposed RF-LS-BPT Model versus Standard RF Modeling Approach

**Figure 6:**
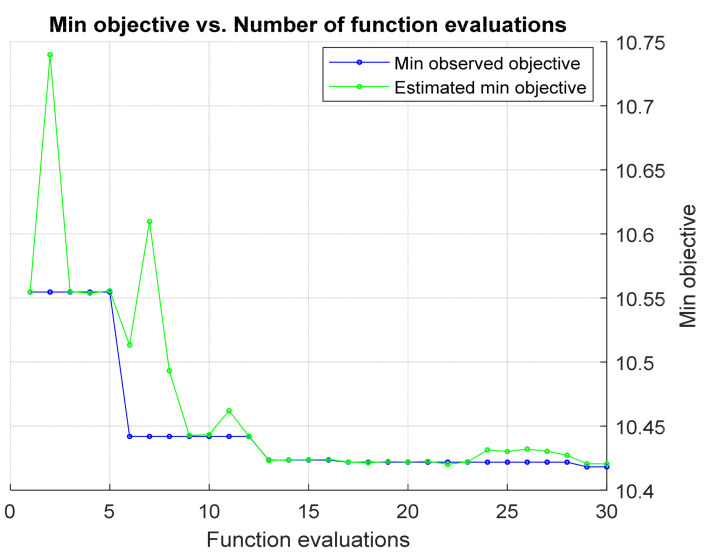
Minimum objective versus function evaluation pattern with Bayesian search.

**Figure 7:**
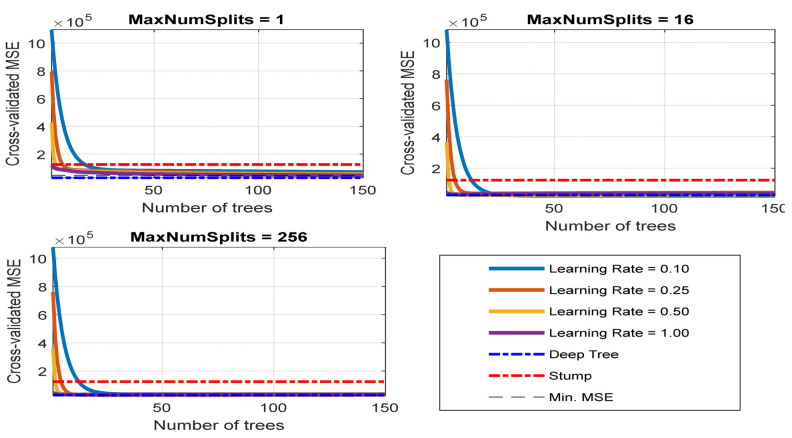
Cross-validated MSE curves arising from hyperparameter tuning.

Although the RF-based regression model outperforms other selected machine learning models, which used benchmarks, as shown above, some hyperparameters can be tuned to further optimize it for improved performance during predictive data modeling and learning. Furthermore, the large prediction error attained by the standard RF-based regression model can be attributed to the high divergence between the input variables and targeted response. As mentioned earlier, the target error response can be reduced using the LS-Boosting technique, hence the proposed RF-LS-BPT model. In order to prevent overfitting or underfitting, the hyperparameters were tuned to minimize the prediction error further [43]. In order to implement the proposed technique, first, a data training set was conveyed through an intended RF regression model empowered with an LS boost ensemble. We then used the Bayesian optimization search-based process to tune and obtain the values of the optimal hyperparameters. The three main focused hyperparameters for tuning are learning rate, number of training cycles (the tree maximum depth), and maxisplits. The same process was repeated, but the Grid search-based hyperparameter tuning method was employed. Figure 6 and Figure 7 display the tuning process patterns and the values of the optimal hyperparameters obtained using grid search and Bayesian optimization search. Notably, the curves in Figure 7 comprise the minimum cross-validated MSE arising after determining optimal hyperparameter values, as shown in Table 3. The table shows the learning rate, tree number, and maximum splits. In order to develop the proposed RF-LS-BPT method, the Bayesian optimization-based search was considered over the grid search since it yielded the lowest error.

**Figure 8:**
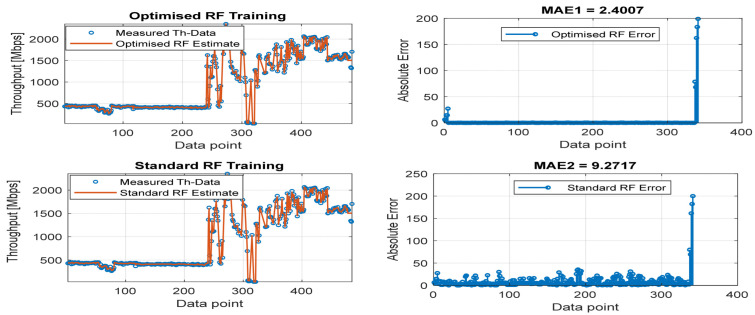
Predicted throughput quality performance of the proposed RF-LS-BPT model and standard RF model at 25 m distance.

**Figure 9:**
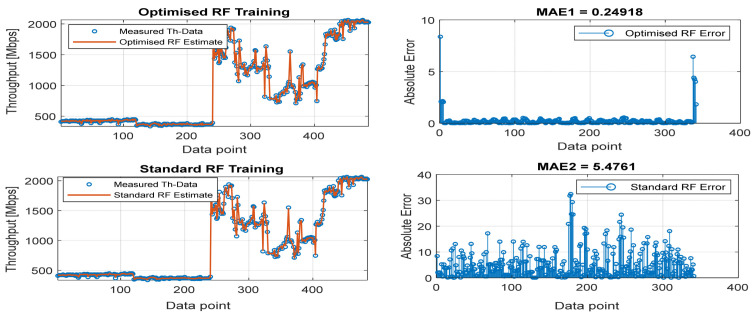
Predicted throughput quality performance of the proposed RF-LS-BPT model and standard RF model at 50 m distance.

**Figure 10:**
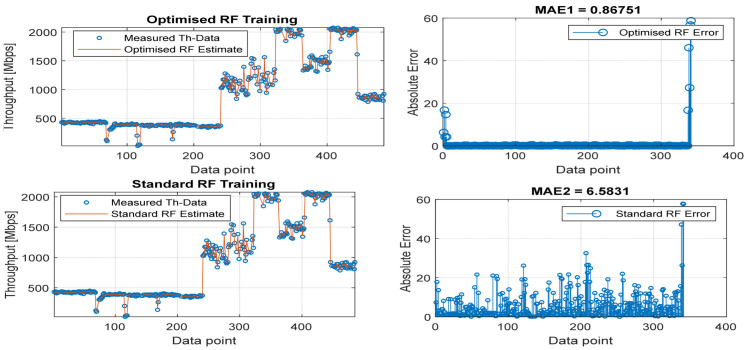
Predicted throughput quality performance of the proposed RF-LS-BPT model and standard RF model at 75 m distance.

**Figure 11:**
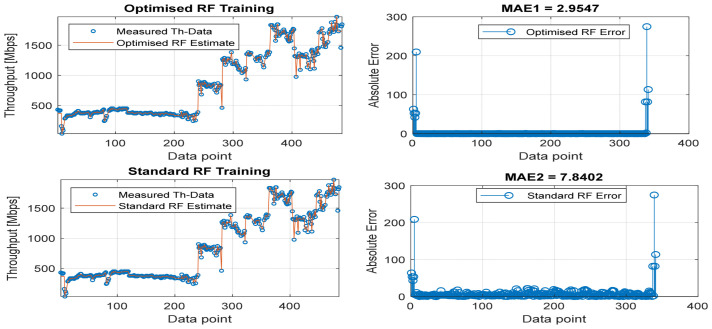
Predicted throughput quality performance of the proposed RF-LS-BPT model and standard RF model at 100 m distance.

**Figure 12:**
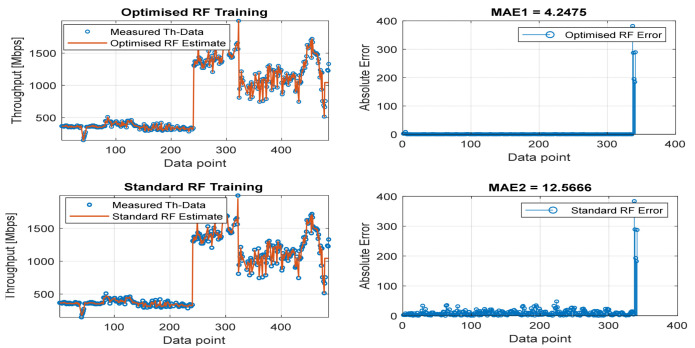
Predicted throughput quality performance of the proposed RF-LS-BPT model and standard RF model at 160 m distance.

**Figure 13:**
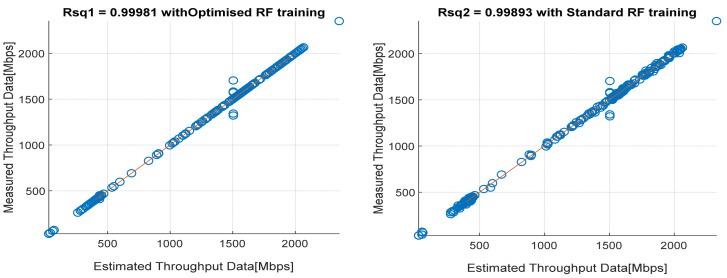
Predicted throughput quality correlation performance of the proposed RF-LS-BPT model and standard RF model at 25 m distance.

**Figure 14:**
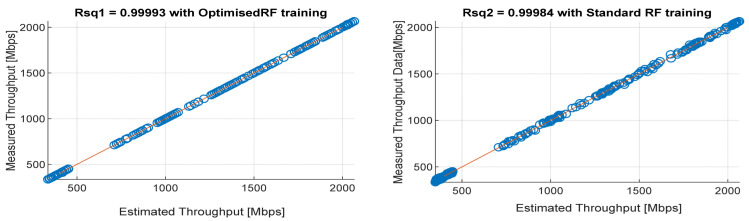
Predicted throughput quality correlation performance of the proposed RF-LS-BPT model and standard RF model at 50 m distance.

**Figure 15:**
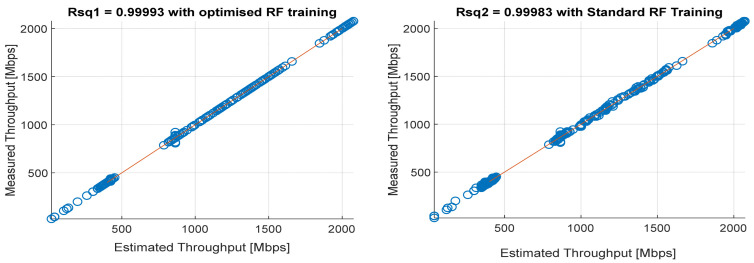
Predicted throughput quality correlation performance of the proposed RF-LS-BPT model and standard RF model at 75 m distance.

**Figure 16:**
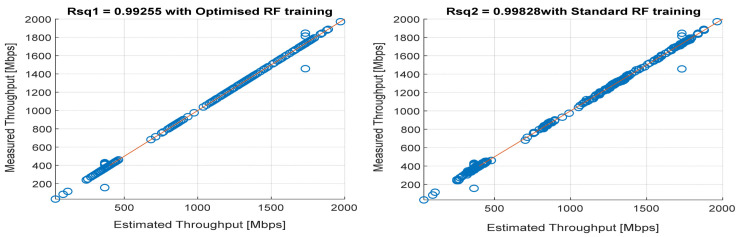
Predicted throughput quality correlation performance of the proposed RF-LS-BPT model and standard RF model at 100 m distance.

**Figure 17:**
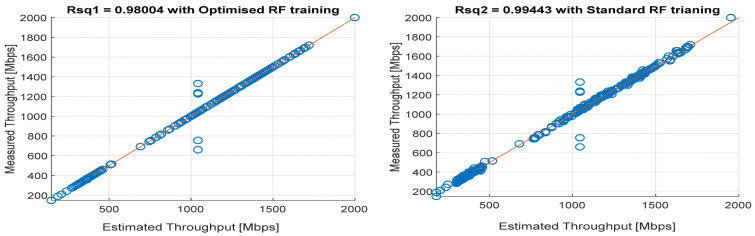
Predicted throughput quality correlation performance of the proposed RF-LS-BPT model and standard RF model at 160 m distance.

Figure 8, Figure 9, Figure 10, Figure 11 and Figure 12 are the results of the proposed RF-LS-BPT regression model. The graphs showing the throughput predictive accuracy attained in MAE values using the proposed RF-LS-BPT method compared to the standard RF approach are shown at different distances. The results show that proposed the RF-LS-BPT method provided the best prediction accuracy. As a case in point, while the proposed method attained 2.40, 0.24, 0.86, 2.95, and 4.29 dB MAE values, the standard RF approach attained poorer throughput predictive accuracy with 9.27, 5.47, 6.58, 7.84, and 12.56 dB MAE values. Similarly, in terms of correlation performance plots, as shown in Figure 13, Figure 14, Figure 15, Figure 16 and Figure 17, the Rsq values attained by the proposed method are in the range of 0.9800 to 0.9999. In contrast, the Rsq values attained by standard RF models are in the range of 0.9644 to 0.9944. Throughput prediction results using key prediction results are summarized in Table 4 and Table 5. These attained throughput prediction performance improvements across the study location show the relevance of the developed RF-LS-BPT regression model over the standard regression approaches.

### 4.4. Throughput Data Training and Testing Using LS-Boosting and Bagging

**Figure 18:**
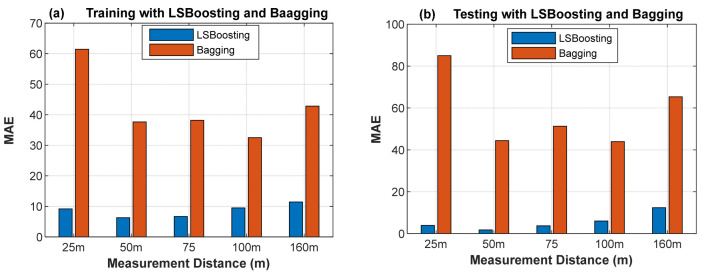
MAE values attained in engaging the LS-Boost and bagging algorithms in the random forest model in adaptively testing the throughput quality at different distances.

Due to hypothetical bias and variance issues, the predicting model or the targeted response may severely suffer from underfitting or overfitting problems, leading to high error between the targeted response and the estimated variables. In order to address such drawbacks, the prediction model needs to be placed under control by employing the bagging (Bag) or least-squares boosting (LS-Boost) algorithm. While bagging employs a simple technique of result averaging to aid a model in achieving its desired prediction, boosting utilizes a weighted mean of results in aiding a model in actualizing its prediction method. In the LS-Boost algorithm, hundreds or more weak learners (trees) are engaged for training, and the error is iteratively updated to improve learning. Here, the robust performance of the adopted LS-Boost algorithm in the proposed RF-LS-BPT model compared to the bagging algorithm in training and testing to learn the throughput data obtained at collection points is provided in Figure 18 using MAE values. Also shown in Table 6 and Table 7 are summaries of accuracy attained by the two RF ensemble algorithms. The robust prediction accuracy achieved in terms of MAE, NRME, and Rsq with the LS-Boost algorithm with the proposed model shows that it helped to considerably improve the extrapolative performance capacity between the targeted throughput data and the estimated variables. Brain and Webb [44,45] opined that models with low bias during learning are generally sought after for large dataset analytics, hence the superiority of our proposed model.

## 5. Conclusions

The throughput quality remained an exclusive higher-layer performance indicator for assessing data transmission quality and integrity in mobile broadband networks. The user data throughput expresses the speed at which a user can reliably send data and receive the same at the user equipment terminal (UET). Generally, the amount and quality of throughput at the UET can fluctuate significantly, subject to many influencing factors. However, many factors can influence user data throughput quality. The key ones include network propagation environment, user location, communication distance, the disproportion between upload and download rates, available channel bandwidth, network traffic load, propagation channel conditions, signal quality, signal coverage, and modulation/coding scheme. The first objective of this research was to determine the actual throughput quality attained at the UET at a close communication distance of 25, 50, 75, 100, and 160 m from the transmitter over a typical 5G mobile broadband cellular network. The second objective was to appraise the popular machine learning predictive modeling techniques in the literature and optimize the best one using a robust approach for optimal adaptive prediction modeling and learning of the acquired stochastic throughput quality. By following the two main objectives, the aims of the proposed learning-based models were achieved. Apart from examining the impact of transmitter–receiver communication distances on throughput quality status as in this study, there is also a need to conduct a detailed empirical investigation of the influence of variables on throughput quality. This need, however, is slated for our future research. In addition, future work would investigate the predictive capability of deep neural network models such as long short-term memory and other evolutionary-based regression techniques such as particle swarm optimization and genetic algorithms.

## Figures and Tables

**Table 1:** Limitations of some related works.

Year	Reference	Focus and Coverage	Limitations	Comparison with This Paper
1989	Battiti [26]	The work focuses on accelerated backpropagation learning, considering two optimization techniques.	There is a need to assess the performance of the models for networks with a large number of weights.	This paper presents a detailed statistical analysis of the acquired throughput data through performance status quality reporting at the different user equipment terminal locations.
2008	Castillo [27]	Adaptive learning algorithms for Bayesian network classifiers were projected. The work aims to handle the cost–performance trade-off and deals with concept drift.	The work did not provide adequate information on how to resolve the bottleneck challenges in a prequential learning framework as the training data increase over time.	The current work examined the performance of the projected learning-based models for 5G wireless networks using large-scale throughput data acquired from several network operators in the United States.
2011	Khan, Tembine, and Vasilakos [28]	The work presents game dynamics and the cost of learning in heterogeneous 4G networks.	The work provides numerical examples and OPNET simulations concerning network selection in WLAN and LTE. However, experimental validation of the numerical results is missing.	Our work presents performance benchmarking of adaptive learning capabilities of different machine-learning-based regression models based on the experimental 5G throughput data.
2016	Pandey and Janhunen [29]	The work presents a method based on reinforcement learning for automating parts of the management of mobile networks.	The work did not cover the concept of learning with partial observability and cooperative learning that considers the neighboring base stations.	Our work addresses the problem of learning with partial observability and cooperative learning by integrating the neighboring base stations based on the 5G data analyzed.
2018	Li, Cao and Hao [30]	The work presents an adaptive-learning-based network selection approach for 5G dynamic environments. The system enables users to adaptively adjust their selections in response to the gradually or abruptly changing environment.	Though the proposed approach enables a population of terminal users to adapt effectively to the network dynamics, experimental validation of the proposed approach is missing.	Our work proposed an RF-LS-BPT regression model for improved dataset predictive modeling and learning based on 5G experimental datasets.
2020	Narayanan et al. [31]	The work focuses on commercial 5G performance on smartphones using 5G networks of three carriers in three US cities. Additionally, the work explored the feasibility of using location and other environmental data to predict network performance.	The work developed practical and sound measurement methodologies for 5G networks on COTS smartphones but did not provide the learning-based models for the 5G performance measurements.	The current work projected learning-based models for improved dataset predictive modeling and learning based on the 5G throughput data.
2021	Moodi, Ghazvini, and Moodi [32]	The work considers a hybrid intelligent approach to detect android botnets using a smart self-adaptive-learning-based PSO-SVM.	The authors observed that one of the factors influencing the selection of important features of a dataset is the approach and the parameters used on that dataset. However, practical deployment of the projected hybrid intelligent approach was not considered.	An optimized RF-LS-BPT regression model was proposed for accurate throughput data modeling and learning using different performance indicators based on experimental datasets.
2022	Hervis Santana et al. [33]	The work examines the application of a machine-learning-based algorithm to approximate a complex 5G path loss prediction model. Specifically, the decision tree ensembles (bagging) algorithm was employed to build a generic model which was used to estimate the pathloss.	Time optimization for the feature (input) calculation process was not considered in this work. Experimental validation of the proposed model is also missing. Lastly, practical testing of the model for accurate wireless network planning is required.	The current work captured optimization for the features (inputs) variables and experimentally validated the proposed model using practical 5G throughput data.

**Table 2:** Throughput quality status attained from 25 to 160 m UET communication distance.

Distance (m)	Max.	Min.	Mean	Median	STD
25	2.35 × 10³	31.43	947.61	450.56	625.27
50	2.08 × 10³	335.54	925.27	734.02	604.43
75	2.07 × 10³	906.13	807.38	807.38	614.36
100	1.97 × 10³	10.49	855.26	718.17	540.10
160	1.99 × 10³	146.79	808.43	655.34	482.53

**Table 3:** Optimal hyperparameter values using grid search and Bayesian search.

Hyperparameters	Best Grid Search Hyperparameter Values	Best Bayesian Search Hyperparameter Values
Learning Rate	0.25	0.29025
Num. Trees	52	23
MaxNumSplits	32	195

**Table 4:** Proposed RF-LS-BPT and standard RF regression model accuracy for training.

	Accuracy	25	50	75	100	160
Optimized RF	MAE 1	2.40	0.42	0.86	2.95	4.24
Standard RF	MAE 2	9.24	5.47	6.58	7.84	12.56
Optimized RF	NRMSE 1	0007	0.0001	0.0027	0.0111	0.081
Standard RF	NRMSE 2	0.009	0.0045	0.0049	0.0117	0.02
Optimized RF	Rsq _21_	0.9999	0.9999	0.9999	0.9986	0.9890
Standard RF	Rsq _22_	0.9998	0.9998	0.9997	0.9983	0.9488

**Table 5:** Proposed RF-LS-BPT and standard RF regression model accuracy for testing.

	Accuracy	25	50	75	100	160
Optimized RF	MAE 1	1.33	0.88	1.12	11.37	11.92
Standard RF	MAE 2	9.27	2.41	3.84	12.88	13.82
Optimized RF	NRMSE 1	0.0041	0.0025	0.0029	0.2700	0.0490
Standard RF	NRMSE 2	0.0043	0.0029	0.0035	0.2720	0.0494
Optimized RF	Rsq _21_	0.9998	0.9999	0.9999	0.9926	0.9881
Standard RF	Rsq _22_	0.9990	0.9977	0.9997	0.9920	0.9800

**Table 6:** Throughput data training accuracy using LS-Boosting and bagging.

	Accuracy	25	50	75	100	160
Training (LS-Boosting)	MAE 1	1.71	0.66	0.72	2.73	5.21
Training (Bagging)	MAE 2	63.03	42.37	37.77	33.57	49.97
Training (LS Boosting)	NRMSE 1	0.0052	0.0012	0.0104	0.0102	0.0210
Training (Bagging)	NRMSE 2	0.0684	0.0500	0.0342	0.0353	0.0560
Training (LS Boosting)	Rsq	0.9996	0.9999	0.9999	0.9986	0.9935
Training (Bagging)	Rsq	0.9835	0.9984	0.9984	0.9883	0.9719

**Table 7:** Throughput data testing accuracy using LS-Boosting and bagging.

	Accuracy	25	50	75	100	160
Testing (LS-Boosting)	MAE 1	4.22	0.71	1.79	8.65	8.07
Testing (Bagging)	MAE 2	77.39	27.91	47.58	43.76	50.08
Testing (LS-Boosting)	NRMSE 1	0.012	0.0024	0.0047	0.024	0.0374
Testing (Bagging)	NRMSE 2	0.090	0.0032	0.0466	0.047	0.0696
Testing (LS Boosting)	Rsq1	0.9983	0.9999	0.9998	0.9947	0.9860
Testing (Bagging)	Rsq2	0.9935	0.9935	0.9935	0.9818	0.9654

## Data Availability

The data that support the findings of this study are publicly available from the University of Minnesota—Twin Cities, USA: https://fivegophers.umn.edu/www20 (accessed on 21 February 2022).

## Abbreviations

4/5/6G	Fourth/fifth/sixth generation
AI	Artificial intelligence
BOA	Bayesian optimization algorithm
Bps	Bits per second
COTS	Commercially available off-the-shelf
DT	Decision tree
EDA	Exploratory data analysis
GLS	Generalized least squares
GN	Gauss-Newton
GP	Gaussian process
GPR	Gaussian process regression
GS	Grid search
KNN	K-nearest neighbor
LM	Levenberg–Marquart
LTE	Long-term evolution
LS	Least squares
LS-Boost	Least-squares boosting
MAE	Mean absolute error
ML	Machine learning
MLP-NN	Multilayer perceptron neural network
NN	Neural network
NRMSE	Normalized root mean squared error
PE	Percentage error
RF	Random forest
RMSE	Root mean square error
RS	Random search
SVM	Support vector machine
UET	User equipment terminal
